# Fabry disease: Mechanism and therapeutics strategies

**DOI:** 10.3389/fphar.2022.1025740

**Published:** 2022-10-26

**Authors:** Xi Li, Xiangyi Ren, Yabing Zhang, Lin Ding, Minfeng Huo, Qian Li

**Affiliations:** ^1^ Department of Anesthesiology, West China Hospital of Sichuan University, Chengdu, China; ^2^ Core Facilities of West China Hospital, Sichuan University, Chengdu, China; ^3^ Shanghai Tenth People’s Hospital, Shanghai Frontiers Science Center of Nanocatalytic Medicine, School of Medicine, Tongji University, Shanghai, China

**Keywords:** fabry disease, lysosomal storage disorder, mechanistic research, fabry therapeutic strategies, alpha-galactosidase A deficiency

## Abstract

Fabry disease is a monogenic disease characterized by a deficiency or loss of the α-galactosidase A (GLA). The resulting impairment in lysosomal GLA enzymatic activity leads to the pathogenic accumulation of enzymatic substrate and, consequently, the progressive appearance of clinical symptoms in target organs, including the heart, kidney, and brain. However, the mechanisms involved in Fabry disease-mediated organ damage are largely ambiguous and poorly understood, which hinders the development of therapeutic strategies for the treatment of this disorder. Although currently available clinical approaches have shown some efficiency in the treatment of Fabry disease, they all exhibit limitations that need to be overcome. In this review, we first introduce current mechanistic knowledge of Fabry disease and discuss potential therapeutic strategies for its treatment. We then systemically summarize and discuss advances in research on therapeutic approaches, including enzyme replacement therapy (ERT), gene therapy, and chaperone therapy, as well as strategies targeting subcellular compartments, such as lysosomes, the endoplasmic reticulum, and the nucleus. Finally, the future development of potential therapeutic strategies is discussed based on the results of mechanistic studies and the limitations associated with these therapeutic approaches.

## 1 Introduction

Lysosomes are organelles that perform a vital function in the catabolism and recycling of cytosolic compounds (Luzio et al., 2007; Boustany, 2013). Deficiencies in proteins with physiological functionalities can lead to lysosomal storage disorders (LSDs) (Schultz et al., 2011; Platt et al., 2018), with over 70 lysosomal enzyme deficiencies causative of LSDs having been identified to date (Martina et al., 2020). Fabry disease is associated with the reduced activity of lysosomal galactosidase A (GLA), an enzyme involved in the catabolism of globotriaosylceramide (Gb3). *GLA* deficiency in patients with Fabry disease results in abnormal glycosphingolipid metabolism and the progressive accumulation of Gb3 (Aerts et al., 2008; Kang et al., 2019). With disease progression, patients display multiple clinical phenotypes, such as cardiovascular disease, neuropathic pain, and even increased risk of early death (Frustaci et al., 2014; Aguiar et al., 2018; Uceyler et al., 2019; Weissmann et al., 2021). However, the cellular pathogenic cascade underlying how *GLA* deficiency results in progressive clinical symptoms remains unclear; moreover, the associated pathogenic cascade has already been initiated before the onset of the organ symptoms of Fabry disease, making *de novo* diagnosis difficult (Jabbarzadeh-Tabrizi et al., 2020; Spinelli et al., 2020). Thus, to achieve adequate therapeutic outcomes and develop novel therapeutic strategies for the treatment of this disorder, a deeper understanding of the associated pathogenic mechanisms is needed.

Several approaches for the treatment of Fabry disease have been exploited, both preclinically and clinically, including enzyme replacement therapy (ERT), gene therapy, and chaperone therapy (Solomon and Muro, 2017; Castelli et al., 2021; Fernández-Pereira et al., 2021). Despite the success of these therapeutic strategies, each approach has its limitations. For instance, the clinical benefit of ERT is limited by the short half-life of the recombinant enzyme, the production of anti-drug antibodies, and the inability of the enzyme to cross the blood-brain barrier (Lenders and Brand, 2018; Abasolo et al., 2021). The recent rapid advances in nanotechnology and nanoscience have provided novel opportunities for overcoming the limitations of current therapeutics (Ghosh et al., 2010; Gregory et al., 2020). The use of biomaterials and nanotechnology enables the development of therapeutic drugs with reasonable pharmacokinetic characteristics, including sufficient blood retention, good bioavailability, and effective immune-evading abilities (Del Grosso et al., 2019; Li et al., 2021). For instance, preclinical and clinical studies have demonstrated that a polyethylene glycol-modified strategy can improve the therapeutic outcome of ERT (Veronese and Pasut, 2005; Schiffmann et al., 2019). Importantly, the development of targeted approaches in nanoscience allows the spatiotemporal control of drug delivery, potentially resulting in high therapeutic efficacy concomitant with a decreased risk of adverse effects.

**FIGURE 1:**
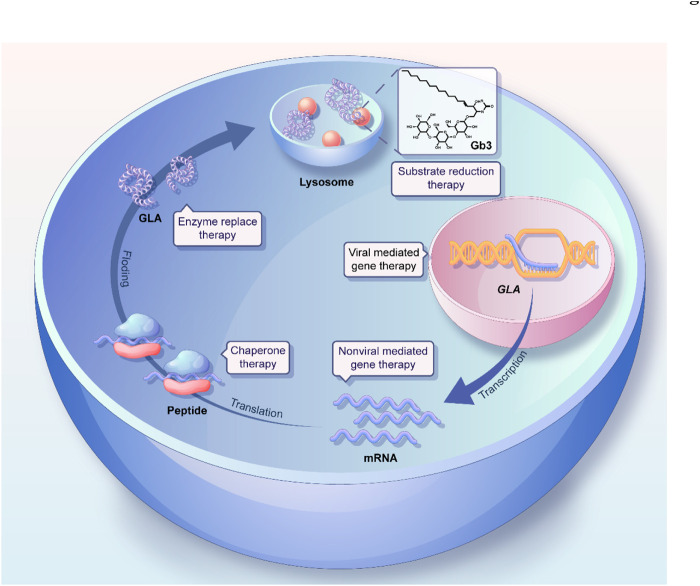
Schematic illustration of the GLA synthetic process and the various therapeutic strategies for Fabry disease.

In this review, we first analyze and discuss recent mechanistic research into Fabry disease, focusing on lysosomal function, autophagy, and lipid metabolism, as well as their connectivity. Subsequently, we systemically summarize the progress in the development of therapeutic modalities for treating Fabry disease, including ERT, gene therapy, and chaperone therapy (Figure 1), as well as the strategies used for the delivery of therapeutic drugs to target sites via the active or passive targeting of nanotherapeutics. In particular, the advances and remaining challenges associated with each therapeutic approach are also addressed. Finally, we describe the promising prospective therapeutic strategies for the treatment of Fabry disease. Overall, we hope that this review will contribute to increasing our understanding of the mechanisms underlying the development of Fabry disease and provide insights regarding current and future therapeutic strategies for the treatment of this disorder.

## 2 Mechanism

### 2.1 Autophagy

Lysosomes are tightly associated with the degradation and recycling of cellular components, including lipids, proteins, and nucleic acids. The inability to digest Gb3 in lysosomes may lead to the activation of several signaling pathways aimed at reducing the levels of accumulated Gb3, eventually resulting in multiple cellular pathologies (Biancini et al., 2012; Hsu et al., 2019). Autophagy plays a critical role in degrading lysosomal substrates (Festa et al., 2018). Several studies have demonstrated that autophagy is upregulated in response to Gb3 accumulation. Gb3 accumulation can activate autophagy pathway to degrade Gb3, alleviating intracellular stresses simultaneously. Although activating autophagy has beneficial effects on lysosomal stress responses in the short term, long-term activation of this pathway can also result in dysregulated autophagy, which accelerates disease progression and worsens pathogenetic changes. Impaired autophagic activity has been observed in peripheral blood mononuclear cells from patients with Fabry disease as well as in several *in vitro* models of Fabry disease (Chévrier et al., 2010; Liebau et al., 2013; Ivanova et al., 2019). For instance, Song et al. constructed a model of Fabry disease in human embryonic stem cells (hESCs) via the CRISPR/Cas9-mediated knockout of the *GLA* gene (Song et al., 2019). The authors found that, compared with their wild-type counterparts, cardiomyocytes differentiated from these hESCs exhibited significantly increased cell size and Gb3 deposition, as well as impaired autophagic flux. These findings indicated that dysregulated autophagy resulting from *GLA* gene deficiency and consequent Gb3 accumulation plays a role in myocardial hypertrophy. In turn, impaired autophagy can further trigger secondary lysosomal deposition via the accumulation of damaged mitochondria and reduced protein degradation, which may further result in a pathogenic cascade and cellular damage. Xing et al. (Xing et al., 2022) demonstrated that impaired autophagic flux can induce myocardial ischemic/reperfusion injury through the accumulation of damaged mitochondria. The restoration of myocardial autophagy improved cardiomyocyte survival *in vitro* and heart function in mice *in vivo*. Combined, these observations suggest that targeting autophagy may represent a potential therapeutic strategy for treating Fabry disease. However, relatively few preclinical or clinical trials have explored the effect of targeting autophagy for the *in vivo* treatment of this disorder. Moreover, the mechanistic basis of the role of autophagy in Fabry disease has not been fully elucidated, and merits further *in vivo* investigation.

### 2.2 Lysosomal dysfunction

The mechanism underlying the Gb3 accumulation-induced intracellular pathogenic cascades remains poorly understood and further studies exploring the link between autophagic disorder and upstream lysosomal functions are necessary. Vacuolar (H^+^)-adenosine triphosphatases (V-ATPases), which use ATP hydrolysis to pump H^+^ into the lysosome, play a central role in maintaining the acidic environment of this organelle (pH = 5.2–6.1) and normal lysosomal function. Lysosomal substrate accumulation in cells leads to increased expression of V-ATPase. AMP-activated protein kinase (AMPK) is also activated in response to depleted ATP levels, and AMPK phosphorylation was observed to be increased in Fabry cardiomyocytes in an *in vitro* model of the disease (Chou et al., 2017). Chou et al. constructed *in vitro* Fabry disease models based on peripheral blood mononuclear cells derived from patients with the disorder (Chou et al., 2017). They found that, compared with controls, Fabry model cells exhibited increased AMPK phosphorylation and elevated glucose transporter 4 (GLUT 4) protein levels, implying that glucose uptake was increased. The authors further investigated energy utilization in Fabry model cells by seahorse metabolic flux assay. Compared with control cells, total energy metabolism, including glycolysis and fatty acid oxidation, was significantly decreased in model cells. Meanwhile, energy metabolism was shifted toward glycolysis from fatty acid β-oxidation. These findings illustrate that *GLA* gene deficiency can induce AMPK activation and enhance glycolysis; however, further *in vivo* investigation is required and aberrant fatty acid metabolism in this disorder needs much deeper exploration. Subsequently, activation of AMPK and V-ATPase can lead to the inhibition of mammalian target of rapamycin complex 1 (mTORC1), which is involved in a variety of signal transduction pathways, including the induction of autophagic activation and lysosomal biogenesis. Interestingly, mTOR activity has been reported to be decreased in Fabry podocytes (Liebau et al., 2013). Combined, the results of these studies suggest that autophagic dysfunction likely results from the triggering of the activation of the AMPK-mTOR signaling pathway due to intracellular Gb3 accumulation. These observations further suggest that the targeting of processes upstream of lysosomes to modulate autophagy may represent an attractive therapeutic strategy for Fabry disease.

### 2.3 Lipid metabolism

An increase in the number of lipid droplets is often seen in Fabry tissues and in *vitro* models of Fabry disease. Recently, Kim et al*.* generated *GLA*-mutant kidney organoids via CRISPR-Cas9 technology. Transmission electron microscopy and oil red O staining analysis showed greater lipid accumulation in Fabry disease model organoids compared with that in the controls (Kim et al., 2021), suggesting that lipid metabolism may have been inhibited in the model organoids. However, the mechanism involved in lipid droplet formation in Fabry disease remains elusive, even in preclinical studies. Disordered fatty acid utilization may underlie the observed lipid droplet formation. First, lipid accumulation may indicate that fatty acid oxidation could be blocked. As discussed above, energy demand is mismatched with the decreasing fatty acid β-oxidation. Defects in carnitine palmitoyl transferase 1 (CPT1), a key enzyme in the transportation of fatty acids into mitochondria for β-oxidation, may lead to lipid formation and dysregulated fatty acid utilization. It has been suggested that increased malonyl-CoA synthesis by acetyl CoA carboxylase can block β-oxidation. Abnormal levels of substrate for fatty acid metabolism are also observed in other Fabry cells (Schumann et al., 2021). LC–MS/MS analysis of acylcarnitines isolated from Fabry disease model cells indicated that the levels of short-chain acylcarnitines and free carnitine were significantly elevated in Fabry cells, whereas those of medium- and long-chain acylcarnitines were decreased. The results of recent studies have confirmed that acylcarnitine accumulation leads to mitochondrial dysfunction and impaired fatty acid oxidation (McCoin et al., 2015; Nguyen et al., 2017). Taken together, these reports suggest that fatty acid metabolism is altered in Fabry disease. However, whether CPT1 has a role in Fabry disease is unknown. Additionally, the link between lipid metabolism and Fabry disease phenotypes requires further elucidation *in vivo*. Secondly, lipid metabolism is strongly associated with autophagy. Under metabolic stress, lipid droplets can be exploited for energy production through autophagy (Rambold et al., 2015; Benador et al., 2019). Blocking autophagy impedes the flux of stored fatty acids from lipid droplets to mitochondria, thus reducing mitochondrial β-oxidation and energy supplementation*.* Another potential explanation for lipid droplets may be related to mitochondrial biogenesis. Peroxisome proliferator-activated receptor (PPAR) and peroxisome proliferator-activated receptor-γ coactivator-1α (PGC1α) are the main regulators of mitochondrial biogenesis via gene transcription. When cellular autophagy is inhibited, the levels of the PPARα repressors histone deacetylase 3 (HDAC3) and nuclear receptor co-repressor 1 (NCoR1) continuously increase as they cannot be degraded in the lysosome (Iershov et al., 2019). Evidence from a preclinical study has indicated that the protein expression of PGC1α is upregulated in Fabry cells (Schumann et al., 2021).

Triacylglycerol (TAG), the major component of lipid droplets, can be reused by mitochondria or removed from cells by high-density lipoprotein (HDL). Interestingly, cholesterol metabolism has been reported to fluctuate in various Fabry disease models as well as in patients with Fabry disease (Shirai et al., 2000; Elliott et al., 2006; Kalliokoski et al., 2006). Some studies have also demonstrated that cholesterol levels are 2∼3-fold higher in Fabry cells than in wild-type cells (Rappaport et al., 2016). Patients with Fabry disease may exhibit compensatory mechanisms for cholesterol dyshomeostasis, such as promoting cholesterol efflux and limiting cholesterol synthesis and uptake. It has been demonstrated that Fabry disease is associated with cholesterol transport, which may contribute to alleviating intracellular stress (Glaros et al., 2005). Gb3 is a constituent of lipoproteins. Once it has been deposited in the lysosome, it must be transported out of the lysosome via intracellular lipid-exporting pathways. Several studies have sought to determine whether there is a potential link between Gb3 and cholesterol homeostasis *in vitro*, with the results showing that the levels of low-density lipoprotein (LDL) are upregulated in glycosphingolipid-treated fibroblasts (Puri et al., 2003; Schueler et al., 2016; van der Veen et al., 2020). Additionally, increasing the levels of LDL can enhance lipid accumulation and facilitate cholesterol influx via cholesterol transport from the circulation (Cedó et al., 2020). Moreover, LDL receptor (LDLR) levels were reported to be increased in cardiomyocytes differentiated from induced pluripotent stem cells of patients with Fabry disease, which contributes to Gb3 storage in vascular cells (Birket et al., 2019). In a retrospective study, total cholesterol and HDL levels were measured before ERT and 10 years after ERT (Stepien and Hendriksz, 2017). Patients who underwent ERT displayed higher HDL levels than those who did not, and ERT was found to (Scharnetzki et al., 2020) exert a cardioprotective effect. These studies indicate that targeting cholesterol metabolism may be a feasible therapeutic option for Fabry disease. In one *in vitro* Fabry disease model, the authors showed that a peptide mimicking the function of apolipoprotein A1 could mediate cellular Gb3 and cholesterol efflux (Schueler et al., 2016). However, a cause-effect link between Fabry disease and cholesterol transport remains elusive, and further studies are needed to allow the development of a therapeutic strategy targeting cholesterol metabolism in Fabry disease.

### 2.4 Inflammatory

Inflammation is a series of adaptive biological responses triggered by several internal or external stimuluses. Controlling inflammatory responses can help restoring host homeostasis and adaptations, while dysregulated inflammatory responses could induce tissue injury and even tissue fibrosis (Medzhitov, 2008; Rozenfeld and Feriozzi, 2017). Progression of Fabry cardiomyopathy is associated with increased inflammatory responses, as demonstrated by elevated inflammatory biomarkers (Yogasundaram et al., 2018). Recently, Song and co-workers reported that pro-inflammation pathway are activated within the cells with Fabry pathology. While the inflammatory responses can be reversed using gene therapy, further confirming the correlation between inflammatory activation and Fabry pathology (Song et al., 2021). Several molecular pathways have been reported to be attributed to the inflammatory activation of Fabry disease, including the nuclear factor kappa B (NF-κB) pathway, oxidative stress, and transforming growth factor-β (TGF-β) pathway. It has also been found that inflammatory activation is closely related to autophagy (defective autophagic protein could enhance inflammasome activation) as well as sphingolipids homeostasis (Levine et al., 2011). Aflaki and co-workers demonstrated that inflammatory response in Gaucher disease is activated through disrupted autophagy (Aflaki et al., 2016). Another study shows that autophagy impairment in Fabry mice exacerbates the pathologies features of renal interstitial fibrosis. Sphingolipids elevate the inflammation responses in many diseases, such as obesity, myocardial infarction, and Parkinson’s disease (Chaurasia et al., 2016; Belarbi et al., 2020; Hadas et al., 2020). For example. Elevated sphingolipids levels (ceramide) were significantly upregulated after myocardial infarction, which triggered severe inflammatory responses and apoptosis of cardiomyocytes (Hadas et al., 2020). Nevertheless, detailed mechanism and consequences about inflammation responses in Fabry disease have not been fully understood. Therapeutic strategy targeting inflammatory to treat Fabry disease is highly demanding and appealing.

## 3 Therapeutic strategies

### 3.1 Enzyme replacement therapy

ERT involves the exogenous supplementation of the GLA enzyme and has been successfully administrated in the treatment of Fabry disease (Pisani et al., 2017). Fabrazyme (α-GLA, *i. v*. 1 mg**·**kg^−1^ every 2 weeks) and replagal (β- GLA, *i. v*. 0.2 mg**·**kg^−1^ every 2 weeks) have both been approved by the United States Food and Drug Administration for the treatment of Fabry disease, and both have shown the potential to significantly reduce Gb3 deposition and improve the prognosis of patients. However, the clinical application of this approach is partly limited by poor biodistribution, a short half-life, and immunogenic responses after intravenous administration (Pisani et al., 2017). For instance, the liver and spleen play a critical role in the rapid clearance of exogenous Gb3, but the biodistribution of heart, kidney, and brain is insufficient to achieve treatment (Desnick and Schuchman, 2012). Additionally, enzymes are easily degraded in the body, while the cost of using ERT is extremely high (∼$200,000 per year). The effect of ERT is also influenced by the treatment starting age. Initiating ERT at an early age (<25 years) can effectively reduce the deposition of Gb3 and attenuate the progressive clinical manifestations of Farey disease (Arends et al., 2017). ERTs did not show a therapeutic benefit in end-stage organ disease or following the production of antibodies in patients (Biegstraaten et al., 2015; van der Veen et al., 2019). This may be explained by the inability of enzymatic therapy to reverse the clinical manifestations. An *in vitro* study demonstrated that treating Fabry podocytes with α-GLA could effectively clear high levels of Gb3 but not restore altered cellular signaling (Braun et al., 2019). Accordingly, addressing the above challenges is crucial for maximizing the therapeutic benefits while concomitantly reducing the harmful effects.

**FIGURE 2:**
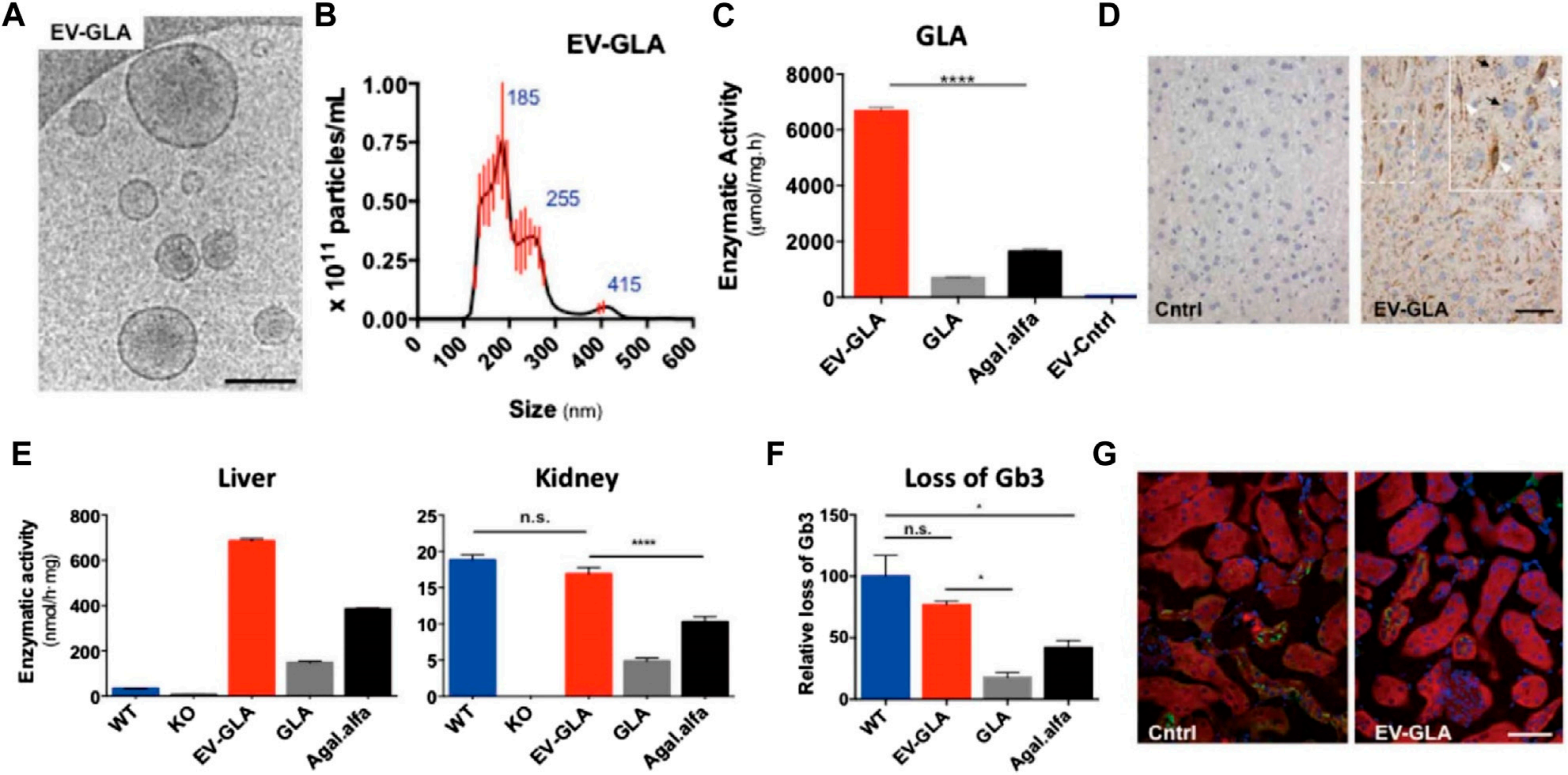
CryoTEM imaging **(A)** and size distribution **(B)** of EV-GLA. Scale bar: 200 nm. **(C)** α-GLA activity test for EV-GLA. **(D)** Immunohistochemistry for the detection of GLA enzyme in liver tissues of GLA knockout (KO) mice after the administration of EV-GLA. Scale bar: 50 μm. **(E)** Measurement of α-GLA activity in the liver and kidney of various groups. **(F)** Measurement of Gb3 loss in various groups. **(G)** Gb3 immunofluorescence (green signal) in the kidneys of KO mice after the administration of a single dose of EV-GLA. Scale bar: 40 μm. Reproduced with permission (Seras-Franzoso et al., 2021). Copyright 2021, The Authors.

Substantial research effort has focused on the development of drug delivery and targeting systems that allow for improved biodistribution and reduced drug clearance (Meka et al., 2016; Gupta et al., 2017). Passive targeting strategies have been proposed focusing on maximizing the effects of ERT (Scaletti et al., 2018). For example, chemical modifications to poly-(ethylene glycol) (PEG) can increase the circulation half-life and reduce the immunogenicity of enzymes in the body (Kizhner et al., 2015). Pegunigalsidase alfa, a PEGylated form of recombinant GLA with a long half-life, has been developed for Fabry disease therapy. *In vivo* pharmacokinetic results demonstrated that the half-life of pegunigalsidase alfa was significantly increased (approximately 10-fold; t_1/2_ = 581 min) compared with that for the commercially available enzyme. Importantly, Fabry male mice treated with PEGylated GLA display reduced anti-drug antibody production. Recently, extracellular vehicles (EVs) derived from GLA-overexpressing cells have been developed as a therapeutic vehicle for the treatment of *GLA* defects (Seras-Franzoso et al., 2021). The EVs not only effectively restored lysosomal function to a greater extent than recombinant GLA, but were also well-tolerated and showed wide organ distribution after *in vivo* administration (Figure 2).

Although nanoparticle passive transport has clear advantages for drug delivery and targeting when compared with free drugs, it is still insufficient. Active targeting has been efficiently exploited to increase the bioavailability of nanoparticles and improve their therapeutic effects. In Fabry disease, active targeting strategies are therapeutically attractive options for achieving high drug bioavailability with reduced loss of enzyme activity (Cabrera et al., 2016; Giannotti et al., 2016; Tomsen-Melero et al., 2021). Arginine-glycine-aspartic acid (RGD) peptides are cyclic peptides that display a high affinity for vascular endothelial cells. Recently, RGD-coated hybrid liposomes conjugated with miristalkonium chloride (MKC) were developed for the loading of GLA for Fabry disease-targeting therapy (Tomsen-Melero et al., 2021). In this design, RGD acts as a targeting moiety by recognizing αvβ3 on endothelial cells. Simultaneously, low concentrations of MKC, a cationic surfactant, enhanced loading efficiency and colloidal stability. Although these nanocarriers improved the bioavailability of therapeutic molecules, the crossing of biological membranes still faces challenges.

Once a therapeutic agent has been delivered to a cell, there are barriers that need to be overcome, including biological barriers of steric hindrance intracellularly to exert its therapeutic effects. Many promising methods have already been developed to address these challenges. Lysosomal-targeting drugs that are released via the lysosomal microenvironment have become one of the primary methods for managing Fabry disease. The lysosomal microenvironment is highly acidic, which enables the release of specific molecules when the nanocarrier is internalized by the lysosome. Cationic materials have been widely applied in the construction of lysosome-targeting nanocarriers. Based on these characteristics, pH-responsive or lysosome-targeting nanomaterials have been developed for the delivery of GLA to the lysosome (Giannotti et al., 2011). Negatively charged GLA was encapsulated into cationic trimethyl chitosan to prepare a pH-responsive nanoparticle. In HMEC-1 cells, the self-assembled nanoparticles exhibited excellent cellular uptake efficiency and pH-triggered release of the GLA enzyme in the lysosome. Besides pH-responsive drug release, lysosomal enzyme-responsive systems represent another favorable approach for targeting delivery GLA. Intercellular adhesion molecule-1 (ICAM-1) is overexpressed in endothelial cells and can specially deliver GLA to ICAM-1-expressing cells. Accordingly, an ICAM-1-targeted nanocarrier (anti-ICAM/α-GLA) was constructed via decorating it with an anti-ICAM antibody (Hsu et al., 2011). In a mouse model of Fabry disease, the administration of the anti-ICAM antibody-coated nanocarrier markedly increased the treatment efficacy of GLA. Numerous strategies using a variety of biomaterials have been employed for lysosomal drug delivery for the treatment of a variety of diseases.

Despite the successes of nanoplatforms in achieving GLA delivery, some critical limitations remain to be overcome. First, the design of GLA-based nanomedicine systems is complicated and should be simplified. Secondly, GLA delivery nanoplatforms for Fabry disease therapy have mainly been established using *in vitro* models. For a better assessment of their therapeutic and clinical translation potential, these nanoplatforms should undergo systemic evaluation in GLA-defective mice, and even in preclinical animal models (e.g., dog, pig, and monkey). Third, the treatment of central nervous system (CNS) manifestations in Fabry disease is hampered by the inability of the enzymes used for ERT to cross the blood-brain barrier; accordingly, strategies that allow these enzymes to access the brain parenchyma to exert their therapeutic effects merit further exploration. Transferrin was recently reported to be able to cross the blood-brain barrier and improve ERT effects through targeting the transferrin receptor that is highly expressed in brain endothelial cells (Ullman et al., 2020). Transferrin may be of great interest in the development of drugs for managing the main manifestations of Fabry disease. Another important issue relates to the biosafety and biocompatibility of nanomedical systems, although advanced nanoscience and materials chemistry can reduce the potential immunogenicity to a certain extent. The long-term biosafety of nanocarriers and materials toxicity of using in synthetic process also need to be considered.

### 3.2 Gene therapy

#### 3.2.1 Viral vector-mediated gene therapy

**FIGURE 3:**
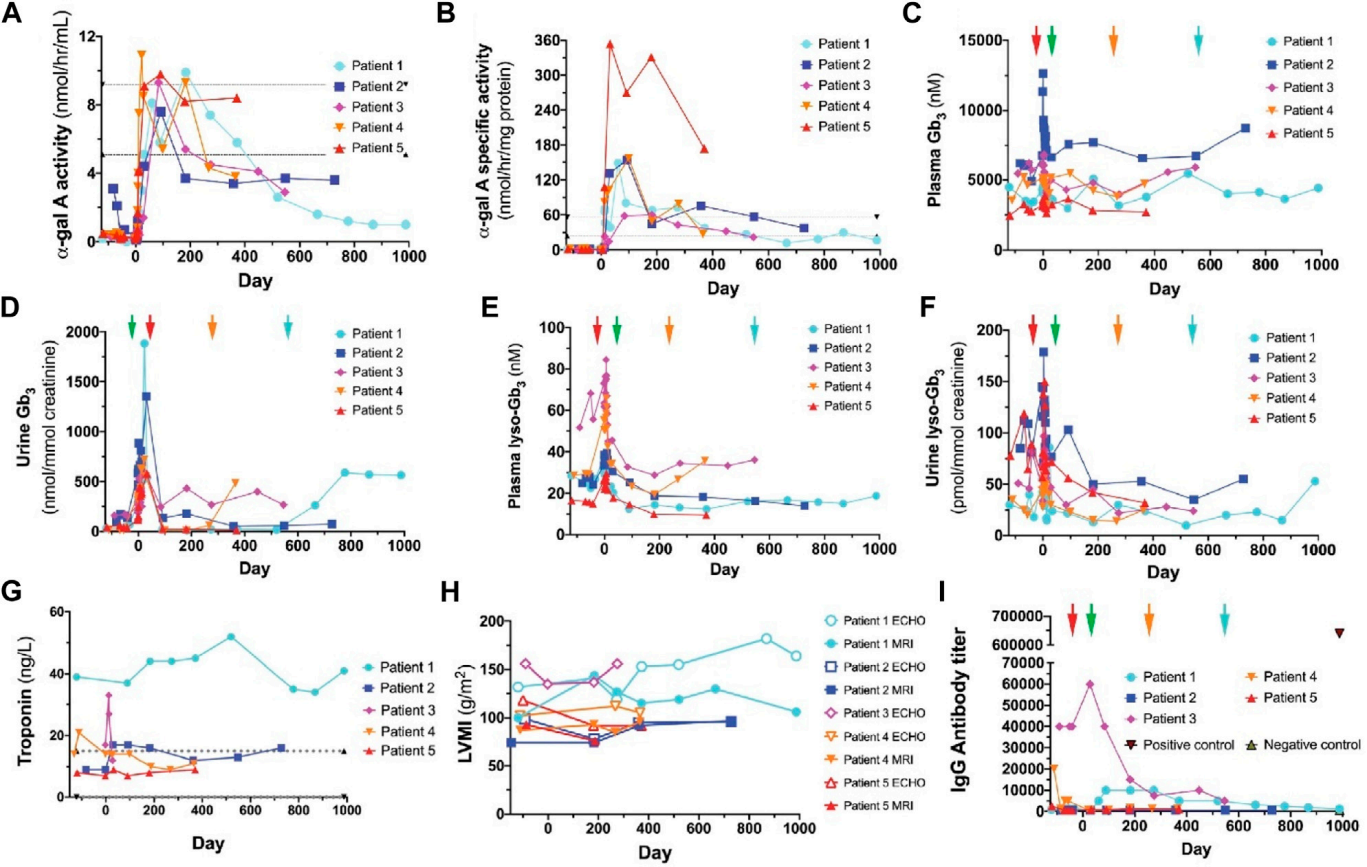
α-GLA activity in plasma **(A)** and leukocytes **(B)** of patients with Fabry disease following gene therapy. The plasma **(C)** and urine **(D)** levels of Gb3 in patients were altered after the initiation of gene therapy. The plasma **(E)** and urine **(F)** levels of lyso-Gb3 were altered after the initiation of gene therapy. Detection of troponin levels **(G)** and left ventricular mass index (LVMI) **(H)** in patients. Patient three did not attend all the measurements. **(I)** Anti-α-GLA antibody levels were assessed in five patients following viral vector-mediated therapy (Khan et al., 2021). Copyright 2021, The Authors.

Gene therapy represents an alternative approach for the treatment of Fabry disease through the application of either viral or nonviral vectors (Yasuda et al., 2020). For viral vector-mediated gene therapy, vectors have been developed that allow the highly-efficient expression of target genes in cells. The advantages of using viral vectors, such as adenoviruses, adeno-associated viruses (AVV), and lentiviruses, include that they can directly correct gene defects and their durable efficacy. Khan et al. demonstrated the effectiveness of lentivirus-mediated therapy in five patients with Fabry disease (Khan et al., 2021). α-GLA activity was significantly increased and Gb3 deposition was reduced in these patients after therapy with hematopoietic stem/progenitor cells expressing α-GLA (Figure 3). The immunogenicity of this system was further analyzed using an enzyme-linked immunosorbent assay (ELISA). Among the five patients who underwent gene therapy, only one displayed increased anti-α-GLA antibody production. The results of this study demonstrated the feasibility of utilizing lentivirus-mediated gene therapy for the treatment of Fabry disease. Despite these promising results, the application of lentivirus-based gene therapy in the CNS is hampered by the presence of the blood-brain barrier. In contrast, several adenoviruses and adeno-associated vectors (AAVs) have been demonstrated to efficiently cross the blood-brain barrier. AAVs have been used to correct *GLA* gene deficiency in the CNS. A recent study explored the therapeutic benefit of AAV9 expressing human α-GLA in Fabry disease model mice (Biferi et al., 2021). After a single intravenous injection, α-GLA activity was significantly increased in several tissues, including the CNS, compared with the control group. A major drawback of this viral vector relates to immunogenicity, random insertion in the host genome, mutagenicity, and organ mistargeting. This highlights the need to take into consideration the safety of viral vectors when designing treatments, thereby facilitating their clinical translation.

Over recent years, attention has increasingly focused on the use of nanotechnology for gene therapy. Some excellent nanocarriers have achieved prominent therapeutic outcomes concomitant with reduced immunogenicity. To avoid antibody-mediated inactivation, PEGylated AAVs have been engineered through the covalent coating of PEG on the surface of the vectors (Lee et al., 2005). Although the circulation time was improved, transduction efficiency was decreased as binding to cells in target tissues was reduced due to the presence of PEG. This observation stresses the need to strike a balance between the circulation time and transduction efficiency. Recently, tannic acid, as a targeting ligand, was used for the modification of AVV9 to achieve myocardial targeting (Shin et al., 2018). Another approach to providing sufficient therapeutic benefits in gene therapy is the accessibility of the cell nucleus for the correction of gene defects. Once delivered to target cells via a nanocarrier, genes must be transported into the nucleus, and targeting therapeutic genes to the nucleus is important for maximizing therapeutic indexes and reducing drug doses. However, viral vectors or nanoparticles often fail to reach the nucleus, which limits their further applicability. To overcome these limitations, nucleus-targeting strategies have been developed, such as the use of nucleus-targeting ligands, including TAT peptide, R8NLS, and Cr10 (Yang et al., 2012; Li et al., 2015; Tu et al., 2020).

#### 3.2.2 Non-viral vector-mediated gene therapy

**FIGURE 4:**
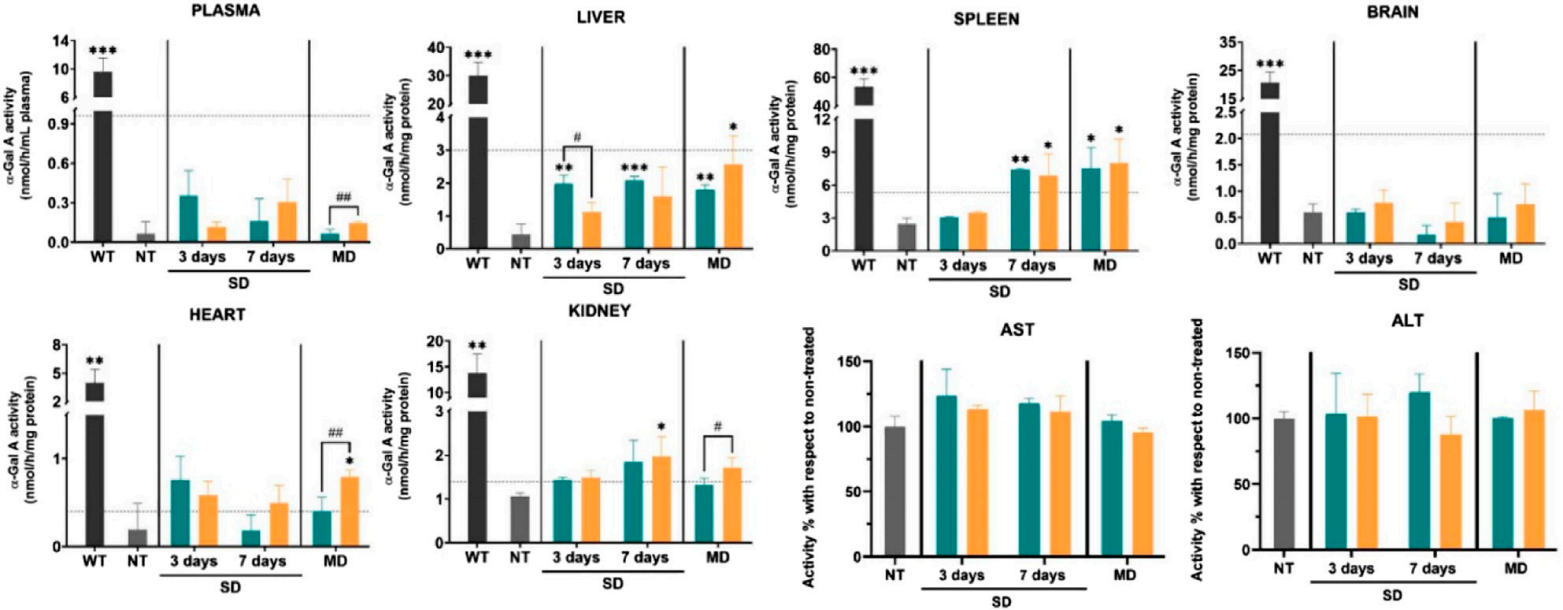
α-GLA A enzymatic activity in the plasma and tissues (liver, spleen, brain, heart, kidney) of different groups and aspartate aminotransferase (AST)/alanine aminotransferase (ALT) activity in GLA knockout (KO) mice after various treatments. NT: non-treated; SD: single dose; MD: multiple doses; Green: plasmid DNA; orange: SLP-based vector. Reproduced with permission (Rodríguez-Castejón et al., 2021). Copyright 2021, The Authors.

Nonviral treatment modalities can be divided into several categories, including siRNA, mRNA, and naked plasmid DNA. Recent studies have reported the therapeutic potential of mRNA in Fabry disease (Ruiz de Garibay et al., 2012; Zhu et al., 2019). Compared with viral vectors, non-viral vectors display substantially less toxicity and immunogenicity. However, the efficacy of systemically administered free mRNA is restricted by its short half-life and instability. Moreover, the low transfection efficacy of non-viral treatment modalities limits their wide clinical application. Successful *in vivo* therapy requires the construction of an RNA delivery system with high stability and reasonable delivery efficiency. This goal can be achieved by designing nanocarriers with high cargo-loading abilities, tissue or organelle specificity, and long circulation times. Numerous attempts have been made to develop a nanocarrier aimed at improving protein expression and RNA stability without systemic adverse effects, such as lipid nanoparticles (LNPs), designed as carriers for the delivery of mRNA for nonviral gene therapy (Rizvi et al., 2021; Tenchov et al., 2021). Encapsulation with LNPs limits RNase-mediated enzymatic degradation of mRNA during the delivery process, leading to increased cellular uptake of mRNA and the production of functional protein. Numerous *in vitro* and *in vivo* studies have confirmed that LNPs have great potential for mediating Fabry disease progression (DeRosa et al., 2019; Zhu et al., 2019). For instance, Aritz et al*.* used solid LNPs to encapsulate the pR-M10-α-GLA plasmid for expressing α-GLA in HepG2 cells (Ruiz de Garibay et al., 2012). LNP encapsulation significantly improved the transfection efficacy and the enzymatic activity of α-GLA. Evidence from a study using Fabry disease model mice also confirmed that solid LNPs can act as carriers for nonviral therapy (Figure 4). (Rodríguez-Castejón et al., 2021) Fabry mice were treated with a non-viral vector (plasmid) based on solid LNPs, and then α-GLA enzymatic activity was evaluated in plasma, liver, spleen, and brain. Mice treated with the GLA-expressing plasmid showed higher α-GLA activity than those treated with the naked plasmid. The biosafety of the LNPs was verified via the measurement of aspartate aminotransferase (AST) and alanine aminotransferase (ALT) activities. These results support that developing nanocarriers for the delivery of nonviral vectors has the potential for markedly improving the therapeutic efficacy of endogenously delivered mRNA.

A key challenge facing the use of LNPs for mRNA delivery lies in that LNPs are mainly confined to hepatocytes, and a lack of cell or organ specificity limits their application (Loughrey and Dahlman, 2022). mRNA taken up by hepatocytes is robustly cleared from the liver, highlighting the importance of improving mRNA delivery to non-liver tissues for systemic mRNA therapy. For Fabry disease, kidney, heart, and CNS are suffering severe pathologies. Therefore, organ targeting and selectiveness are of critically importance for effective mRNA therapy (Knott et al., 2019). To satisfy such demand, LNP-based systems that specifically transport mRNA to specific tissues or organs are urgently demanded. Various LNPs with structural features and biological properties have been developed for organ-targeted mRNA delivery (Li et al., 2019). Notably, LNPs can be modified with various targeting ligands, including active targeting ligands and stimuli-responsive groups. For example, LNPs with RGD modifications can precisely bind vascular endothelial cells, thus improving therapeutic efficacy and reducing off-targeted effects. Moreover, specific organ-targeting LNPs have been developed to facilitate precise mRNA delivery to different organs for treatment (Dilliard et al., 2021; Qiu et al., 2022). These observations demonstrate that targeting specific organs may represent a potential strategy for improving LNP-based mRNA delivery for the treatment of Fabry disease.

### 3.3 Chaperone therapy

Another clinically available approach for the treatment of Fabry disease is chaperone therapy, which involves the stabilization of the conformation of GLA protein in the endoplasmic reticulum to improve its catalytic ability (Riccio et al., 2020). Chaperone therapy can be divided into three types based on the associated mechanism, namely, exogenous competitive chaperones, exogenous non-competitive chaperones, and endogenous molecular chaperones (Suzuki, 2021). Compared with ERT, chaperone therapy has the advantages of excellent bioavailability, oral administration, and the ability to cross the blood-brain barrier (Pereira et al., 2018). Migalastat, an α-galactosidase analog, represents a typical exogenous competitive chaperone for Fabry disease therapy. The efficacy of migalastat has been demonstrated in preclinical studies and patients with Fabry disease. Jonas et al*.* explored the therapeutic effect of migalastat in patients with Fabry disease and reported that, after 1 year of oral administration of migalastat, the patients showed increased α-GLA levels and reduced serum creatinine concentrations (Müntze et al., 2019). Meanwhile, the myocardial mass index was significantly improved, further demonstrating the efficacy of migalastat. It is generally believed that restoring α-GLA enzymatic activity to over 10% may effectively maintain normal physiological function and ameliorate clinical manifestations in Fabry patients (Parenti et al., 2015). Despite its successful application in the clinic, the benefits of chaperone therapy vary among patients owing to the fluctuating levels of residual enzymatic activity. Migalastat is considered to be an appropriate therapeutic option for the treatment of Fabry patients with amenable mutations.

**FIGURE 5:**
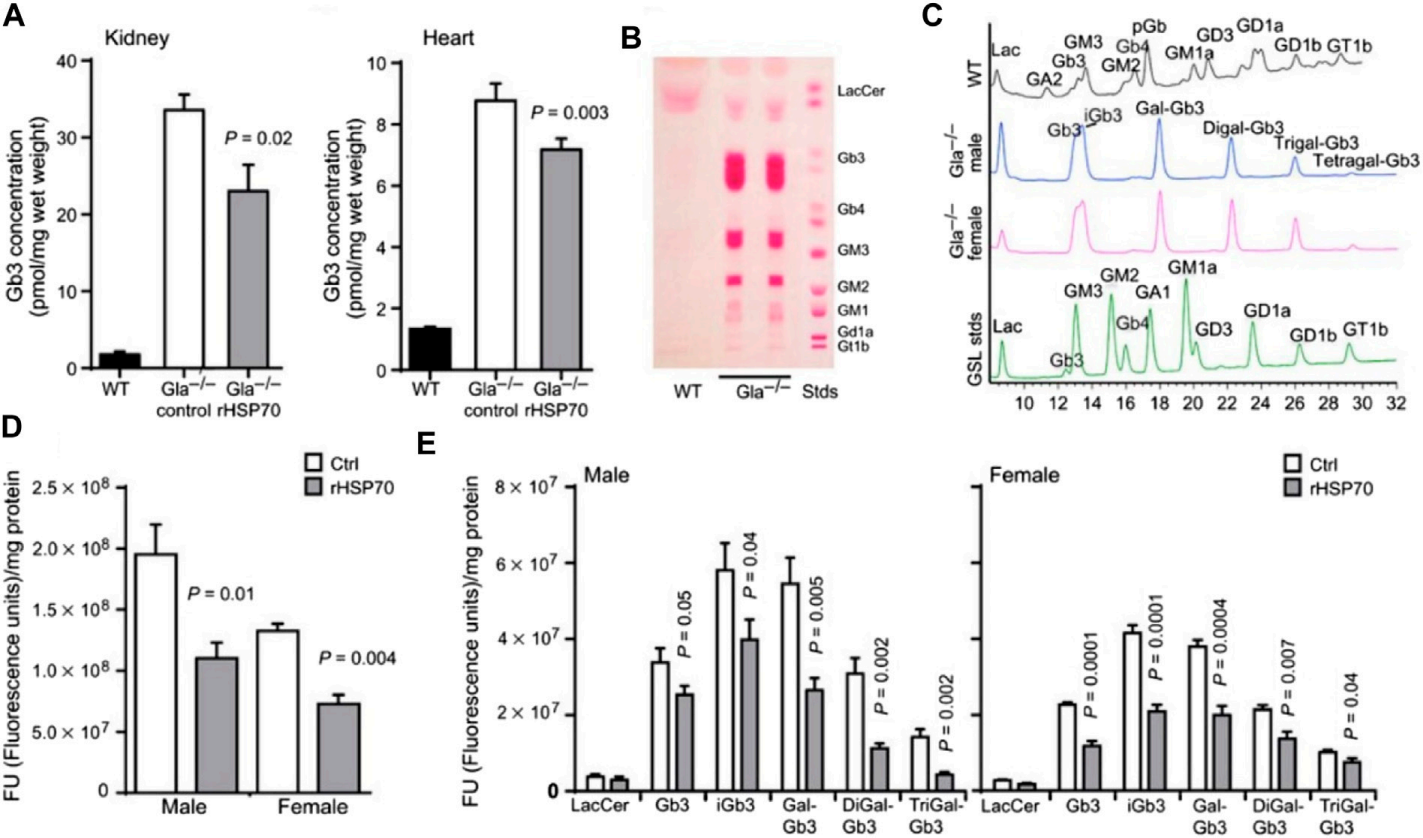
**(A)** Quantification of Gb3 concentrations in the heart and kidneys after various treatments. **(B)** Thin-layer chromatographic **(B)** and high-pressure liquid chromatographic **(C)** analysis of glycosphingolipid storage in wild-type (WT) and GLA knockout (KO) mice. Quantification of glycosphingolipid contents in dorsal root ganglia of KO mice with various treatments. Ctrl: control, PBS; rHSP70: rHSP70 intraperitoneal injection, 5 mg**·**kg^−1^. Reproduced with permission (Kirkegaard et al., 2016). Copyright 2016, American Association for the Advancement of Science.

Heat shock proteins (HSPs) also show promise as small-molecule therapeutic chaperones. HSPs comprise a family of endogenous chaperone proteins that play multiple roles in response to cellular stress, including facilitating protein folding. HSPs can be classified into several groups according to their molecular weight, which ranges from 12 to over 100 kDa. Importantly, HSPs are important mediators of various diseases, including cancer, myocardial infarction, and ischemic stroke (Kumar et al., 2016; Behdarvandy et al., 2020; Wu et al., 2020). Additionally, HSP-based strategies have been developed as therapeutic interventions for the treatment of lysosomal storage diseases. Recombinant human HSP 70 was recently reported as having the capacity to alleviate sphingolipid storage in various lysosomal storage diseases by binding lysosomal bis(monoacylglycero)phosphate to sphingolipid-degrading enzymes and thereby enhancing sphingolipid catabolism (Kirkegaard et al., 2016). In primary fibroblasts from patients with Fabry disease, co-incubation with HSP 70 for 24 h can effectively improve the function of lysosomal-related enzymes, thus effectively alleviating sphingolipid accumulation. Meanwhile, in Fabry disease model mice, glycosphingolipid levels in the kidney, heart, and dorsal root ganglion were significantly reduced following the administration of 5 mg**·**kg^−1^ recombinant HSP 70 (Figure 5). These studies provide new insight into HSP-mediated chaperone therapy in Fabry disease. The Chaperone therapy/ERT combination may provide therapeutic advantages over chaperone therapy alone or ERT alone. An enzyme active-site stabilizer (D-galactose-configured a-cyclosulfamidate) has been reported to improve the therapeutic effect of Fabrazyme (Artola et al., 2019). In an *in vitro* cell model, fibroblasts from Fabry patients co-treated with Fabrazyme and a-cyclosulfamidate displayed significantly enhanced enzyme activity and decreased Gb3/LysoGb3 accumulation. These improved therapeutic effects were ascribed to competitive inhibition of α-GLA and the stabilization of its conformation. These results suggest that combination therapies may provide greater therapeutic benefits than monotherapies in the treatment of Fabry disease.

Artificial chaperones that mimic the functions of natural chaperones have been developed for biomedical applications, such as the treatment of tumors and Alzheimer’s disease (Hashimoto et al., 2018; Li et al., 2020; Ma et al., 2020). Several nanomaterials have been designed to achieve chaperone functions. For example, Yang et al*.* constructed a self-assembling micelle to mimic HSP 70 function by combining poly (-amino ester: hydrophobic microdomains)-block-poly (ε-caprolactone) and poly (ethylene oxide: hydrophilic chain segment)-block-poly (ε-caprolactone). The hydrophobic microdomains could selectively capture amyloid-beta (Aβ) and prevent its aggregation in a mouse model of Alzheimer’s disease (Yang et al., 2019). The concept of artificial chaperones has also been used in facilitating the refolding of lysosomal glycoside hydrolases (Nakamoto et al., 2016). In another report, Kawasaki et al. constructed an artificial nanogel chaperone using polysaccharide and iron oxide nanoparticles to assist β-GLA delivery and folding (Kawasaki et al., 2016). These results suggest that artificial chaperones may provide a feasible alternative to current options in the treatment of Fabry disease.

The endoplasmic reticulum (ER) is where most protein folding occurs, and targeting the delivery of drugs to the ER has been applied in biomedicine (Shi et al., 2021). Shiga toxin comprises a toxic A subunit and a nontoxic B subunit that can specifically bind to Gb3. Therefore, Shiga toxin conjugated medications can be used for ER delivery through Gb3 targeting (El Alaoui et al., 2007). Targeting the ER via Shiga toxin B represents a novel avenue for the treatment of Fabry disease. Apart from Shiga toxin, other compounds such as sulfonyl-containing ligands and sulfunol-peptides can be complexed with nanocarriers to target the ER (Ting et al., 2014; Fang et al., 2019).

### 3.4 Substrate reduction therapies

Substrate reduction therapy (SRT) refers to the application of molecular medications to reduce the storage of undigested macromolecules by inhibiting the synthesis of the corresponding substrates (Marshall et al., 2019). The advantages of SRT lie on the high bioavailability and its capability in traversing blood-brain barrier. Nevertheless, high residual enzyme activity is typically necessary for effective SRT. Recently, the development of medications for reducing Gb3 accumulation is receiving increasingly attentions in lysosomal storage disease (e.g., Gaucher, Fabry, and GM1-gangliosidosis disease) (Fischetto et al., 2020; Kok et al., 2021). For instance, Lucearstat and Venglustat have been clinically available for Fabry disease to reduce Gb3 accumulations. Lucearstat (N-butyl deoxy galactono-jirimycin) is a molecular inhibitor to competitively inhibit glucosylceramide synthase that catalyzes ceramide into glucosylceramide. The tolerability, biosafety, and efficacy of this medication in treatments against Fabry disease have been investigated in a clinical trial experiment (NCT02930655). Patients with Lucearstat treatment were observed with decreased plasma Gb3 concentration and relieved organ pathologies. Biosafety of Lucearstat has also been validated. These trials show that Lucearstat holds great potentials for clinical treatment of Fabry disease. In addition, phase three clinical trials are focusing the therapeutic effect and management of Fabry neuropathic pain (NCT03425539). Unfortunately, phase three clinical result show that Fabry patient subjected with 6-month Lucearstat treatment fail to alleviate the neuropathic pain of Fabry patients. For Venglustat, another promising SRT drug operated by inhibiting glucosylceramide synthase and reducing Gb3 production. Phase two clinical trials of Venglustat have validated its potential in treatment against Fabry disease (NCT02228460). Currently, trials on long-term effectiveness and safety are on the way (NCT02489344).

## 4 Conclusion and outlook

The mechanism underlying Gb3-induced organ dysfunction in Fabry disease remains largely unknown. Further studies on the fundamental mechanism involved in the pathophysiology of Fabry disease is expected to lead to improved strategies for its management. Autophagy provides new insights of how Gb3 accumulation and autophagic disorder benefit disease treatments and drug development. In this regard, the use of autophagic inhibitors may represent a promising avenue for the treatment of Fabry disease via reducing secondary lysosomal accumulation. Additional research efforts are required to reveal the therapeutic benefits of autophagic inhibitors. Previous evidence has revealed that activation of the AMPK-mTOR pathway has an important role in the modulation of autophagy. Thus, targeting processes upstream of the lysosomal signal for managing Fabry disease has shown great potential in preclinical studies. Interestingly, some studies have established a connection between Fabry disease and lipid hemostasis, which may provide new insights into disease progression and innovative therapies. Nevertheless, the therapeutic benefits of modulation of lipid hemostasis and inflammatory response have not been demonstrated, even in preclinical studies. Fundamental efforts are needed to further clarify the role of cholesterol metabolism and inflammatory activation in Fabry disease.

With the progress of nanotechnology and nanoscience, the limitation of these modalities can be partly solved. Designing a lysosome-based subcellular-targeted nanoparticle may be a feasible strategy for disease intervention. This targeting strategy has resulted in excellent therapeutic outcomes in lysosomal storage disease by targeting nanoparticles to the lysosome and subsequently triggering high pH-dependent drug release. Several lysosomal targeting delivery systems have been developed in experimental studies. Nevertheless, issues associated with systemic application limit their clinical translation potential. Many medications are still premature for intracellular localization. Therefore, tissue targeting design should be considered prior to the lysosomes targeting design. Accordingly, combining subcellular targeting with active targeting may be an effective strategy for treating Fabry disease. Another constraint to achieving targeted and responsive therapy relates to the complexity of synthetic processing, which can influence the biosafety of nanocarriers. Thus, it is necessary to design simpler nanoplatforms to achieve sufficient therapeutic effects without long-term systemic toxicity.
